# Mutual Arrangements of Coronary Blood Vessels within the Right Atrial Appendage Vestibule

**DOI:** 10.3390/jcm10163588

**Published:** 2021-08-15

**Authors:** Jakub Hołda, Katarzyna Słodowska, Marcin Strona, Karolina Malinowska, Filip Bolechała, Katarzyna A. Jasińska, Mateusz Koziej, Katarzyna Piątek-Koziej, Jerzy A. Walocha, Mateusz K. Hołda

**Affiliations:** 1 HEART—Heart Embryology and Anatomy Research Team, Department of Anatomy, Jagiellonian University Medical College, 31-034 Cracow, Poland; jakub.p.holda@gmail.com (J.H.); kslodow11@gmail.com (K.S.); karolamali@wp.pl (K.M.); k.jasinska@uj.edu.pl (K.A.J.); m.koziej@uj.edu.pl (M.K.); k.piatek@uj.edu.pl (K.P.-K.); j.walocha@uj.edu.pl (J.A.W.); 2 Department of Forensic Medicine, Jagiellonian University Medical College, 31-034 Cracow, Poland; m.strona@uj.edu.pl (M.S.); f.bolechala@uj.edu.pl (F.B.); 3 Department of Cardiovascular Sciences, University of Manchester, Manchester M13 9PL, UK

**Keywords:** right atrial appendage, right atrial appendage vestibule, right atrium, ablation, cardiac anatomy, right coronary artery

## Abstract

Background: The aim of our study was to investigate the presence and mutual relationships of coronary vessels within the right atrial appendage (RAA) vestibule. Methods: We examined 200 autopsied hearts. The RAA vestibule was cross-sectioned along its isthmuses (superior, middle, and inferior). Results: The right coronary artery (RCA) was present in 100% of the superior RAA isthmuses but absent in 2.0% of hearts within the middle isthmus and in 6.5% of hearts within the inferior RAA isthmus. Its diameter was quite uniform along the superior (2.6 ± 0.8 mm), middle (2.9 ± 1.1 mm), and inferior (2.7 ± 0.9 mm) isthmuses (*p* = 0.12). The location of the RCA varied significantly, and it was sometimes accompanied by other accessory coronary vessels. In all the isthmuses, the RCA ran significantly closer to the endocardial surface than to the epicardial surface (*p* < 0.001). At the superior RAA isthmus, the artery was furthest from the right atrial endocardial surface and this distance gradually decreased between the middle RAA isthmus and the inferior RAA. Conclusions: This study was the most complex analysis of the mutual arrangements and morphometric characteristics of coronary blood vessels within the RAA vestibule. Awareness of additional blood vessels within the vestibule can help clinicians plan and perform safe and efficacious procedures in this region.

## 1. Introduction

The vestibule of the right atrial appendage (RAA) is an endocardial area of the right atrium located between the orifice of the right atrial appendage and the right atrio-ventricular valve (RAV) annulus [1,2,3]. The RAA vestibule is intersected by three isthmuses: an inferior, middle, and superior isthmus. The wall of the vestibule is composed of endocardial, myocardial, and adipose tissue layers that have varying degrees of thickness at different levels [4].

The area of the RAA vestibule has several uses in clinical medicine. It is often a site for radiofrequency ablations, valvular device implantation, or RAV repair procedures [5,6]. Some innate anatomical features of the region may impede the success of these interventions. For instance, coronary vessels present within the wall of the vestibule (especially the right coronary artery and cardiac veins) and in proximity to the endocardial surface can interfere with ablation procedures. These vessels are at risk of physical injury, compression, kinking, occlusion, thrombosis, and/or thermal injury [7]. Although rare, procedures performed within the area of the RAA vestibule can cause serious complications such as atrial wall rupture or cardiac tamponade [8,9]. Therefore, a thorough understanding of the morphology and of the spatial relationships of coronary vessels within the targeted region may prevent adverse procedural outcomes.

Only a few studies have examined the course of the coronary vessels within the area of the RAA vestibule, and most of them focused on analyzing the trajectory of the right coronary artery [3,10]. A study by Al-Ammouri et al. analyzed 10 pediatric hearts and measured the distance between the artery and the endocardial surface [10]. Ueda et al. tracked the course of the right coronary artery along the RAA vestibule in 44 autopsied human hearts [3]. Taken together, the data from these studies could not convey a complete image of blood vessels within the RAA vestibule. Therefore, the aim of this study was to provide an accurate assessment of the dimensions and the various interrelationships of coronary vessels within the RAA vestibule. We hope that our findings will influence clinical interventions and make them safer and more effective.

## 2. Materials and Methods

This study was approved by the Bioethical Committee of the Jagiellonian University in Cracow, Poland (No 1072.6120.90.2020). The study protocol conforms to the ethical guidelines of the 1975 Declaration of Helsinki.

### 2.1. Study Population

This study examined 200 adult human hearts (Caucasian) of both sexes (22.0% female). The subjects were between the ages of 18 and 94 (mean = 46.9 ± 17.9 years). The average body mass index (BMI) of the donors was 26.6 ± 4.5 kg/m^2^, and the average body surface area (BSA) was 1.9 ± 0.2 m^2^. The hearts were collected during routine forensic medical autopsies. The primary causes of death included suicide, traffic accidents, murder, and home accidents. Donors with severe anatomical defects, heart trauma, heart grafts, and severe cardiovascular macroscopic pathologies and those with signs of cadaveric decomposition were excluded. None of the donors died of heart failure, and none had a known history of arrhythmias.

### 2.2. Dissection and Measurements

The chest cavities of the donors were opened in a routine manner. The hearts were dissected with the adjacent parts of the large vessels: the aorta, the pulmonary trunk, the superior vena cava, the inferior vena cava, and the pulmonary veins. After dissection, the hearts were weighed using a 0.5 g precision electronic laboratory scale (SATIS, BSA-L Laboratory, Ostrów Wielkopolski, Poland). They were then preserved in a 10% paraformaldehyde solution through passive immersion.

To access the RAA vestibule, the right atrial wall was cut in between the superior vena cava and the ostium of the inferior vena cava. Inside the RAA vestibule, three distinct isthmuses were distinguished: the inferior RAA isthmus (located at the inferior/terminal crest end of the RAA vestibule), the superior isthmus (located at the superior/septal end of the RAA vestibule), and the middle isthmus (located halfway between the inferior RAA isthmus and the superior RAA isthmus). The height of each isthmus was recorded. To obtain their cross section, each isthmus was cut longitudinally, perpendicular to the endocardial surface.

Each of the obtained cross-sections was additionally divided into 3 equal sectors: an upper sector (close to the RAA orifice), a lower sector (close to the RAV annulus), and a middle sector (found in between the upper and lower sectors). All blood vessels within the cross sections were identified along with their tissue layer location (ex: myocardial vs. adipose tissue layer). A flexible probe was inserted into each vessel to determine its course and origin. The diameters of the lumina of the coronary vessels were measured. Additional parameters were obtained from the right coronary artery running within the RAA vestibule. These included:

- The shortest distance from the margin of the right coronary artery to the endocardial surface of the right atrium;
- The shortest distance from the margin of the right coronary artery to the margin of the RAA orifice;
- The shortest distance from the margin of the right coronary artery to the margin of the RAV annulus;
- The thickness of the whole right atrial at the level of the artery (spanning from the endocardium to the epicardium).

All linear measurements were obtained using 0.03 mm precision electronic calipers (YATO, YT–7201, Wrocław, Poland). Measurements were performed by two independent researchers to reduce human bias. If results reported by the two researchers differed by more than 10%, the sample was reassessed. The mean of the two new measurements was calculated and approximated to the tenth decimal place.

### 2.3. Statistical Analysis

The data are presented as the mean ± the standard deviation (SD). For continuous variables, the data are presented as the median with the corresponding interquartile range (Q1, Q3), whereas categorical variables are presented as a number (in percentage (%)). The Shapiro–Wilk test was used to determine if the quantitative data were normally distributed. Continuous parameters were compared using the Mann–Whitney U test or nonparametric analysis of variance (Kruskal–Wallis) test. Analysis of variance (ANOVA) was used to compare values between different isthmuses. Correlation coefficients were calculated to measure the statistical dependence between various heart features. To detect a simple correlation (*r* = 0.25) with 80% power and a 5% significance level (two-tailed; α = 0.05; β = 0.2), the minimal sample size was calculated to be 123 cases. The chi-squared test was used for categorical data comparison. We performed statistical analyses with STATISTICA 13.1 (StatSoft Inc., Tulsa, OK, USA). A *p* value of less than 0.05 was considered a statistically significant finding.

## 3. Results

### 3.1. Superior RAA Isthmus

**Figure 1:**
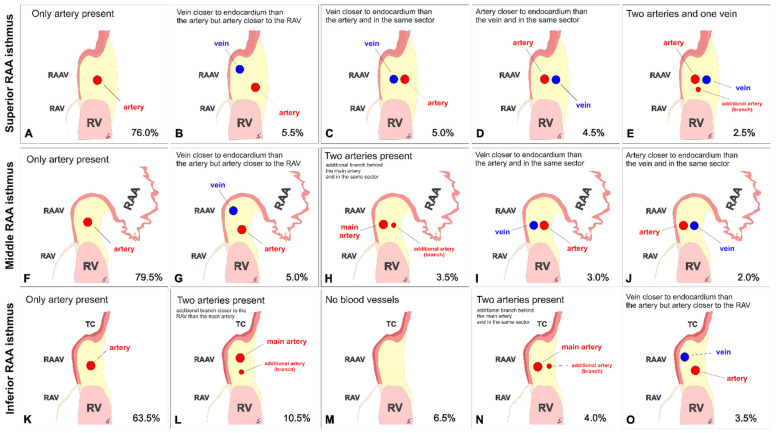
Schematic view of the cross sections thorough right atrial appendage (RAA) isthmuses. Five most common types of spatial relations between coronary vessels within the superior (**A**–**E**), middle (**F**–**J**), and inferior (**K**–**O**) RAA isthmuses are present. RAAV—right atrial appendage vestibule, RAV—right atrio-ventricular valve, RV—right ventricle, TC—terminal crest.

**Figure 2:**
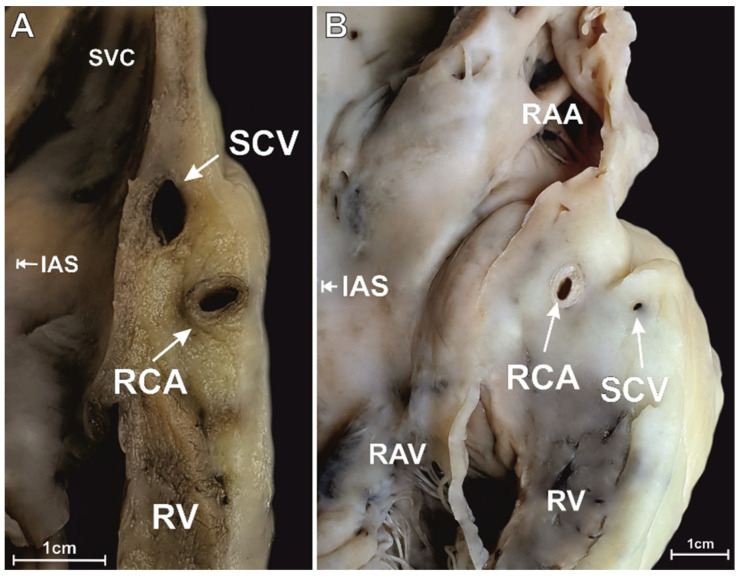
Photographs of a cadaveric heart specimen showing the longitudinal section through the superior right atrial appendage (RAA) isthmus. Cases with two main coronary vessels in dif-ferent arrangements are presented. (**A**) vein closer to the endocardium than the artery but closer to the RAV. (**B**) artery closer to the endocardium than the vein and in the same sector. IAS—interatrial septum, RAV—right atrio-ventricular valve, RCA—right coronary artery, RV—right ventricle, SCV—small cardiac vein, SVC—superior vena cava.

At the level of the superior RAA isthmus, the right coronary artery was present in every single examined heart (100%). Other distinct vessels were found in 24.0% of studied specimens. The small cardiac vein (a tributary of the coronary sinus) was seen in 6.5% of cases, whereas the anterior cardiac vein (that enters the right atrium directly) was noted in 10.0% of cases. An additional coronary artery (the acute marginal branch) was seen in 5.0% of hearts, whereas 2.5% of specimens had both an additional vein and artery. The different configurations of the coronary vessels within the superior RAA isthmus are shown in Figure 1A–E. The interposed coronary artery (located between the coronary vein and the right atrial endocardial surface) was found in 7.0% of hearts (Figure 1D,E). Most arteries and veins were located in the adipose tissue layer (98.5% and 97.5%, respectively). The remaining vessels were situated within the myocardial tissue (Figure 2). The right coronary artery was predominantly located in the lower sector of the isthmus (closer to the valve annulus) (66.5% of hearts). Other locations of the right coronary artery included: the middle sector of the isthmus (28% of cases); the upper sector of the isthmus (3.5% of cases). The artery was located at the level of the RAV annulus in 2.5% of hearts and below the annulus in 0.5% of cases; in 23.5% of hearts, it was located closer than 2.0 mm from the annulus. Right coronary arteries located less than 2.0 mm from the endocardium were present in 3.0% of cases. Table 1 presents the morphometric characteristics of the right coronary artery within the superior RAA isthmus. The mean diameter of the small cardiac vein was significantly smaller than the diameter of the right coronary artery (0.8 ± 0.3 vs. 2.6 ± 0.8 mm, *p* < 0.001). The acute marginal branch also had a smaller diameter than the right coronary artery (1.9 ± 0.5 vs. 2.6 ± 0.8 mm, *p* = 0.001). The thickness of the entire right atrial wall at the level of the artery was 17.9 ± 6.5 mm, and it did not differ when other vessels were present (*p* > 0.05).

### 3.2. Middle RAA Isthmus

**Figure 3:**
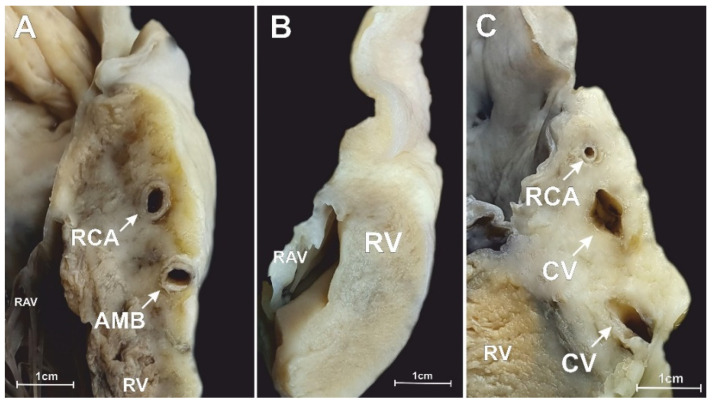
Photographs of a cadaveric heart specimen showing the longitudinal section through the middle right atrial appendage isthmus. (**A**) The right coronary artery (RCA) with acute marginal branch (AMB) is present. (**B**) Case with no coronary vessels present within the isthmus section. (**C**) RCA accompanied by two coronary veins (CV). RAV—right atrio-ventricular valve, RV—right ventricle.

In the middle RAA isthmus, the right coronary artery was present in 98.0% of hearts (it was not found in 2.0% of cases). Other vessels were found in 18.5% of specimens. One single coronary vein (the small cardiac vein) was visible in 10.0% of cases. The acute marginal branch was observed in 7.0% of cases, whereas a combination of accessory vessels was seen in 1.5% of cases (Figure 3). The spatial relationships of the coronary vessels within the middle RAA isthmus are shown in Figure 1F–J. The interposed coronary artery was found in 2.5% of hearts (Figure 1J). Similar to the superior RAA isthmus, most arteries and veins were located in the adipose tissue layer (although two arteries and one vein were located within the myocardial tissue). The right coronary artery was predominantly located in the middle sector of the isthmus (47.5% of cases). Other locations of the artery included: the upper sector of the isthmus (34.5% of cases); the lower sector of the isthmus (10.5% of cases). The artery was located at the level of the RAV annulus (3.5% of cases) and below the RAV annulus (2.0% of cases); in 7.0% of hearts, it was located closer than 2.0 mm from the annulus. Right coronary arteries located less than 2.0 mm from the endocardium were present in 4.5% of cases. Table 1 presents the morphometric characteristics of the right coronary artery within the middle RAA isthmus. 

The additional vessels found within the middle RAA had smaller diameters than the right coronary artery (small cardiac vein: 1.1 ± 0.4 vs. 2.9 ± 1.1 mm, *p* < 0.001 and acute marginal branch: 1.8 ± 0.6 vs. 2.9 ± 1.1 mm, *p* < 0.001). The thickness of the entire right atrial wall at the level of the artery was 13.5 ± 4.4 mm, and it did not differ when other vessels were present (*p* > 0.05).

### 3.3. Inferior RAA Isthmus

**Figure 4:**
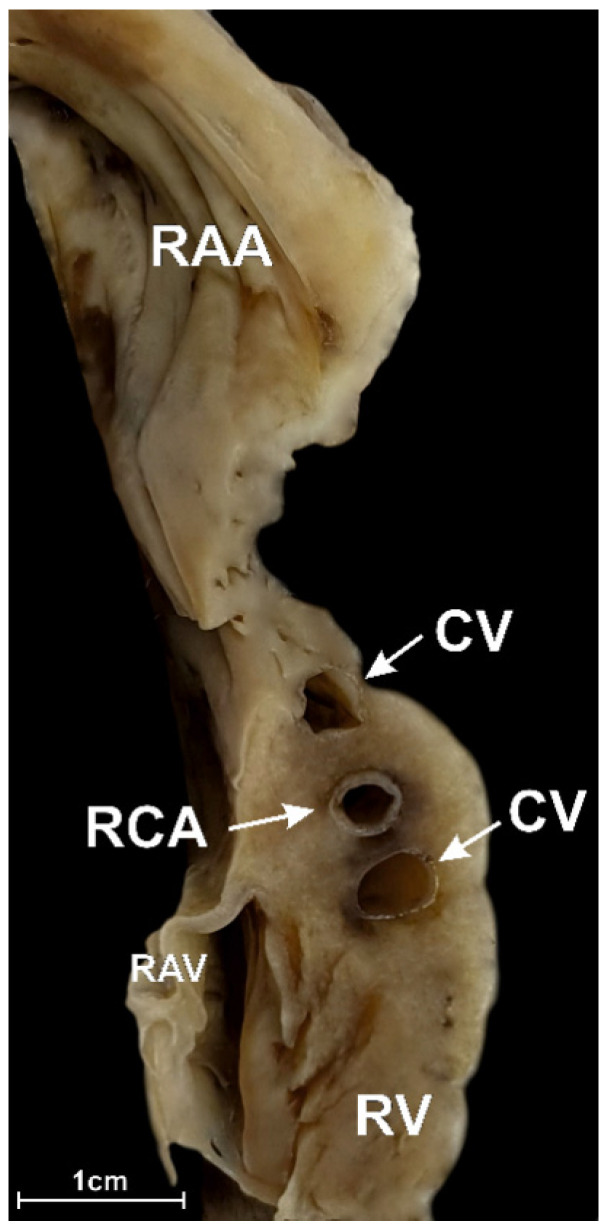
Photograph of a cadaveric heart specimen showing the longitudinal section through the inferior right atrial appendage (RAA) isthmus with right coronary artery (RCA) accompanied by two coronary veins (CV). RAV—right atrio-ventricular valve, RV—right ventricle.

In the inferior RAA isthmus, no coronary vessels were found in 6.5% of examined hearts. The right coronary artery was present in 93.5% of hearts. Other vessels were found in 30.0% of specimens. The acute marginal branch was seen in 18.5% of cases. The small cardiac vein was seen in 7.5% of hearts, whereas multiple accessory veins or arteries were noted in 4.0% of cases (Figure 4). The different configurations of the coronary vessels within the inferior RAA isthmus are shown in Figure 1K–O. The interposed coronary artery was found in 1.5% of hearts. Most arteries and veins were located within the adipose tissue layer, and only 1.0% of vessels were found within the muscular layer of the isthmus. The right coronary artery was predominantly located in the middle sector of the isthmus (43.0% of cases). Other locations of the artery included: the lower sector of the isthmus (23.5% of cases); the upper sector of the isthmus (19.0% of cases). The artery was located at the level of RAV annulus in 6.5% of hearts and below the annulus in 1.5% of cases; in 14.0% of hearts, it was located closer than 2.0 mm from the annulus. Right coronary arteries located less than 2.0 mm from the endocardium were present in 6.0% of cases. Table 1 shows the morphometric characteristics of the right coronary artery within the inferior RAA isthmus. Similar to the superior RAA isthmus, the additional vessels found within the middle RAA had smaller diameters than the right coronary artery (small cardiac vein: 1.7 ± 0.4 vs. 2.7 ± 0.9 mm, *p* < 0.001 and acute marginal branch: 1.8 ± 0.4 vs. 2.7 ± 0.9 mm, *p* < 0.001). The thickness of the entire right atrial wall at the level of the artery was 9.1 ± 2.8 mm, and it did not differ when other vessels were present (*p* > 0.05).

### 3.4. The Morphometry of the Right Coronary Artery within the Isthmuses

The right coronary artery was present in all the examined superior RAA isthmuses. It was absent in 2.0% of hearts within the middle RAA isthmus and in 6.5% of hearts within the inferior RAA isthmus. Its diameter was quite uniform along the superior, middle, and inferior isthmuses (Table 1, *p* = 0.12). The location of the artery varied significantly. Within the superior RAA isthmus, it lay in close proximity to the RAV anulus (the mean distance between the artery and the valve annulus was 3.9 ± 2.9 mm). Along its course, it deviated towards the RAA orifice (see Table 1, *p* < 0.001). Moreover, the distance between the right coronary artery and the endocardial surface differed significantly when comparing the three isthmuses. In all of them, the artery ran closer to the endocardial surface than the epicardial surface (Table 1, *p* < 0.001). At the superior RAA isthmus, the artery was furthest from the right atrial endocardial surface, and this distance gradually decreased between the middle RAA isthmus and the inferior RAA isthmus (9.0 ± 4.0 vs. 6.2 ± 3.0 vs. 4.8 ± 2.3 mm, respectively, *p* < 0.001) (Table 1). The same trend was observed for the distance between the right coronary artery and the epicardial surface (6.4 ± 4.4 vs. 4.3 ± 3.1 vs. 2.2 ± 1.6 mm, respectively, *p* < 0.001). The right coronary artery was located less than 2.0 mm from the epicardial surface in 1.0% of cases within the superior RAA isthmus, 3.5% of cases within the middle RAA isthmus, and in 6.0% of cases within the inferior RAA isthmus. Moreover, the thickness of the atrial wall gradually thinned out along the course of the right coronary artery (17.9 ± 6.5 vs. 13.5 ± 4.4 vs. 9.1 ± 2.8 mm, respectively, *p* < 0.001). 

There were no age or sex variables linked to vessel location within all the RAA isthmuses. Vessels dimensions were not affected by any anthropometric parameters (sex, age, weight, height, BMI).

## 4. Discussion

The spatial relationships between individual components of the coronary vasculature are not fully understood, and knowledge on this subject is based on the analysis of individual cases rather than on research conducted in accordance with the principles of evidence-based anatomy [11]. The results of this study are consistent with the findings presented by Ueda et al. [3]. However, the much larger sample size, the higher number of morphometric factors analyzed, and the documentation of additional vessels present within the RAA vestibule make this work unique. The right coronary artery was found in the overwhelming majority of RAA isthmuses; however, its prevalence differed between isthmuses (it was found in 100% of the superior isthmuses, in 98.0% of the middle isthmuses, and in 93.5% of the inferior RAA isthmuses). The right coronary artery that traversed alongside the RAV annulus had a diameter that varied slightly along its course through the vestibule, although this trend was not found to be statistically significant. The distance between the endocardium and the right coronary artery gradually decreased to less than 5 mm in the lower segments of the RAA vestibule. In the upper RAA vestibule segment, the artery was surrounded by an abundant adipose tissue pad. However, in this segment the artery was located at the closest distance to the valve annulus when compared with the middle and inferior RAA isthmuses (Table 1).

The presence of the right coronary artery may have a significant impact on procedures performed within the RAA vestibule. Understanding the relationship between the RAV annulus and the surrounding blood vessels is of great importance for procedure optimization [12]. The presence of the artery carries the risk of its own injury (wall damage, occlusion, compression, kinking, intraluminal thrombosis, or acute spasm). This in turn can cause serious consequences such as cardiogenic shock and electrical instability, which may lead to death [7,13,14,15]. It was proven that artery interposed between the vein and endocardial surface is a predictor of unsuccessful linear ablation during endocardial and epicardial ablation. In those cases, an injury to the artery is almost certain when performing the epicardial ablation from the coronary venous system. Previous studies have shown that the abovementioned complications are particularly applicable to vessels running close to the endocardial surface and closer than 2 mm from the hinge lines of the RAV leaflets [12,16]. Our study showed that right coronary arteries located less than 2 mm from the endocardium were relatively rare, although such occurrences were more frequent within the inferior RAA isthmus (6.0% of cases). Along with the fact that the right coronary artery was located closer than 2.0 mm from the valve annulus in 14.0% of cases within the inferior RAA isthmus, it would be likely that the lower segments of the RAA vestibule carry the greatest risk of injury.

Furthermore, it is important to realize that arteries may act as heat-sinks. The blood flow inside the vessels is responsible for the local cooling of the right atrial myocardium, and this may decrease the effectiveness of ablation procedures within the RAA vestibule [17]. Two factors play a key role in the heat-sink effect—the distance to the vessel and its diameter [18,19]. This study showed that the diameter of the right coronary artery was quite uniform along its course through the RAA vestibule, but its proximity to the endocardial surface varied significantly—it was closest to the endocardium at the level of the inferior RAA isthmus. Moreover, the acute marginal branch was most frequently observed within the inferior RAA isthmus. Its presence could provide an accessory escape route for energy supplied during ablation procedures. Thus, the cooling heat-sink effect would be most significant within the lower segments of the RAA vestibule.

Up until now, no study had provided information on the presence and location of any additional vessels within the RAA vestibule. Our study shows that within the RAA vestibule, vessels other than the coronary artery were relatively rare. The small cardiac vein was present in only 6.5% of hearts within the superior RAA isthmus, in 10.0% of hearts in the middle isthmus, and in 7.5% of hearts in the inferior RAA isthmus. Its diameter was also always smaller than that of the right coronary artery. For these reasons, the small cardiac vein should not be considered as an adequate access site for epicardial ablations or electrophysiological mapping within the area of the RAA vestibule [20]. On the other hand, clinicians should not be wary of additional veins being present because their iatrogenic damage would not lead to serious complications. Regardless, it is possible to avoid this dilemma by visualizing the small cardiac vein prior to procedures via high-row coronary computed tomography angiography [21].

It is worth mentioning that the wall of the RAA vestibule itself may also be prone to high risk of injury (and thus perforation) during invasive procedures, especially when recesses or sectional wall thinning occur. Moreover, we should remember about the risk of damaging other structures lying in the RAA vestibule area. The right phrenic nerve with accompanying vessels (pericardiophrenic artery and vein) is one of the structures that may be injured during catheter ablation procedures [22].

Our study had several limitations. Foremost, it was clearly a macroscopic study based on autopsied and paraformaldehyde fixed tissue. Therefore, minor changes in cardiac size and shape may have occurred due to fixation. However, our previous experimental studies have shown that the use of 10% paraformaldehyde did not cause significant changes in the diameters of blood vessels and did not significantly affect the dimensions of atrial tissue [23,24]. Second, since we analyzed postmortem material, we were not able to assess the behavior and dimensional changes of the studied area within the cardiac cycle or the blood flow (hemodynamics) in the vessels. Moreover, we examined hearts from healthy donors, and we do not know if our findings could be applicable to patients with structural heart disease or other cardiac pathologies. Finally, we were not able to indicate any ethnic differences due to the ethnic homogeneity of our studied population (Caucasian). Despite these limitations, we deem that they did not significantly affect the topographical and morphological analyses of the relationships between coronary vessels and their relative dimensions within the RAA vestibule.

## 5. Conclusions

The right coronary artery is the most frequently occurring vessel within the vestibule of the RAA isthmus. Its presence was noted in 100% of hearts within the superior isthmus, 98.0% of hearts within the middle isthmus, and in 93.5% of hearts within the inferior RAA isthmus. The small cardiac vein was observed in the superior isthmus in 6.5% of cases, in the middle isthmus in 10.0% of cases, and in the inferior RAA isthmus in 7.5% of cases. The location of the coronary vessels varied significantly between isthmuses. Variables such as age or sex or anthropometric features did not affect the morphometric characteristics of coronary blood vessels within the RAA vestibule. Awareness of the existence of other blood vessels within the RAA vestibule area can help clinicians prepare and perform safe and efficacious interventions.

## Figures and Tables

**Table 1:** Dimensions of the right coronary artery (RCA) identified within the right atrial appendage (RAA) isthmuses and its relations to the endocardial surface, right atrioventricular valve annulus (RAVA), and RAA orifice.

Parameter	Superior RAA Isthmus			Middle RAA Isthmus			Inferior RAA Isthmus		
	Mean ± SD	Range (Min–Max)	Median (Q1; Q3)	Mean ± SD	Range (Min–Max)	Median (Q1; Q3)	Mean ± SD	Range (Min–Max)	Median (Q1; Q3)
RCA diameter (mm)	2.6 ± 0.8	0.9–4.8	2.0; 3.1	2.9 ± 1.1	0.7–6.7	2.4; 3.8	2.7 ± 0.9	0.6–6.0	2.1; 3.3
RCA to RAVA distance (mm)	3.9 ± 2.9	(−0.5)–29.7	2.1; 5.0	6.2 ± 3.4	(−6.0)–16.8	4.1; 8.4	5.0 ± 3.2	(−3.5)–17.6	3.0; 6.7
RCA to RAA orifice distance (mm)	10.3 ± 3.7	0.5–23.3	7.7; 12.4	5.5 ± 3.8	0.0–23.5	2.2; 8.0	5.7 ± 3.2	0.1–14.8	3.2; 8.1
RCA to endocardial surface distance (mm)	9.0 ± 4.0	0.5–21.9	6.2; 11.3	6.2 ± 3.0	1.2–18.1	4.2; 7.7	4.8 ± 2.3	0.6–11.9	3.1; 6.1
RCA to epicardial surface distance (mm)	6.4 ± 4.4	0.5–27.8	3.4; 8.6	4.3 ± 3.1	0.4–16.7	1.7; 6.4	2.2 ± 1.6	0.2–7.9	0.9; 2.9

SD—standard deviation; Q1 and Q3—lower and upper quartiles.

## Data Availability

Data are available from the authors upon reasonable request.
